# Altered humoral immunity to mycobacterial antigens in Japanese patients affected by inflammatory demyelinating diseases of the central nervous system

**DOI:** 10.1038/s41598-017-03370-z

**Published:** 2017-06-09

**Authors:** Davide Cossu, Kazumasa Yokoyama, Yuji Tomizawa, Eiichi Momotani, Nobutaka Hattori

**Affiliations:** 1 Juntendo University School of Medicine, Department of Neurology, Tokyo, 113-8421 Japan; 2 Tohto College of Health Sciences, Department of Human-care, Saitama, 366-0052 Japan

## Abstract

*Mycobacterium avium subsp. paratuberculosis* (MAP) and *Mycobacterium bovis* (BCG) have been associated to several human autoimmune diseases such as multiple sclerosis (MS), but there are conflicting evidence on the issue. The objective of this study is to evaluate their role in Japanese patients affected by inflammatory demyelinating disorders of the central nervous system (IDDs). A total of 97 IDDs subjects including 51 MS and 46 neuromyelitis optica spectrum disorder (NMOSD) patients, and 34 healthy controls (HCs) were tested for the detection of IgG, IgM and IgA against mycobacterial antigens by indirect ELISA. The levels of anti-MAP IgG were higher in MS patients compared to NMOSD patients (AUC = 0.59, p = 0.02) and HCs (AUC = 0.67, p = 0.01), and the anti-MAP antibodies were more prevalent in MS patients treated with interferon-beta (OR = 11.9; p = 0.004). Anti-BCG IgG antibodies were detected in 8% of MS, 32% of NMOSD and 18% of HCs, the difference between MS and NMOSD groups was statistically significant (AUC = 0.66, p = 0.005). Competition experiments showed that nonspecific IgM were elicited by common mycobacterial antigens. Our study provided further evidence for a possible association between MAP and MS, while BCG vaccination seemed to be inversely related to the risk of developing MS.

## Introduction

Multiple sclerosis (MS) is the most common inflammatory demyelinating disease (IDDs) of the central nervous system (CNS) and it is mainly caused by T cells reactive against components of myelin^1^. Neuromyelitis optica spectrum disorder (NMOSD) is characterized by the development of recurrent optic neuritis and/or longitudinally extensive transverse myelitis^2^. Astrocytopathy and secondary demyelination is mediated by antibodies (Abs) targeting aquaporin 4 (AQP4) protein, but exist also a variant AQP4-negative such as myelin oligodendrocyte glycoprotein (MOG) positive. The origins of the pathogenic autoimmune attack in MS and how the Abs against AQP4 appear in NMOSD are not known, and the pathogenesis of both diseases results from complex interactions between genetic and environmental factors^3^. Different studies pointed out the possibility that one or more infectious pathogens might trigger autoimmunity^4^, and the immune response against *Mycobacterium avium* subsp. *paratuberculosis* (MAP) and *Mycobacterium bovis* strain bacille Calmette-Guérin (BCG) has been associated with several human diseases such as MS, however, their role in the pathologic process has been controversial and sometimes opposite^5, 6^.

In two recent studies conducted on MS and healthy Japanese subjects, a statistically significant percentage of MS patients resulted Ab-positive against MAP_2694_295-303_ peptide^7^ and MAP surface antigens^8^. This finding highlighted the possibility that Japanese could be exposed to MAP antigens, and a small fraction of these people might be genetically susceptible to the development of autoimmune disorders^8^. On the other side, BCG vaccine seems to have a beneficial effect on MS development. Different clinical trials proved that BCG vaccination may be able to reduce the magnetic resonance imaging activity in patients with relapsing-remitting (RRMS) and clinically isolated syndrome (CIS) in Italy^6^.

For this reason, we aimed to evaluate for the first time the humoral response to different mycobacteria in Japanese MS patients compared to NMOSD and healthy controls (HCs).

## Results

### Anti-MAP IgG Ab-titer is increased in MS patients

**Figure 1:**
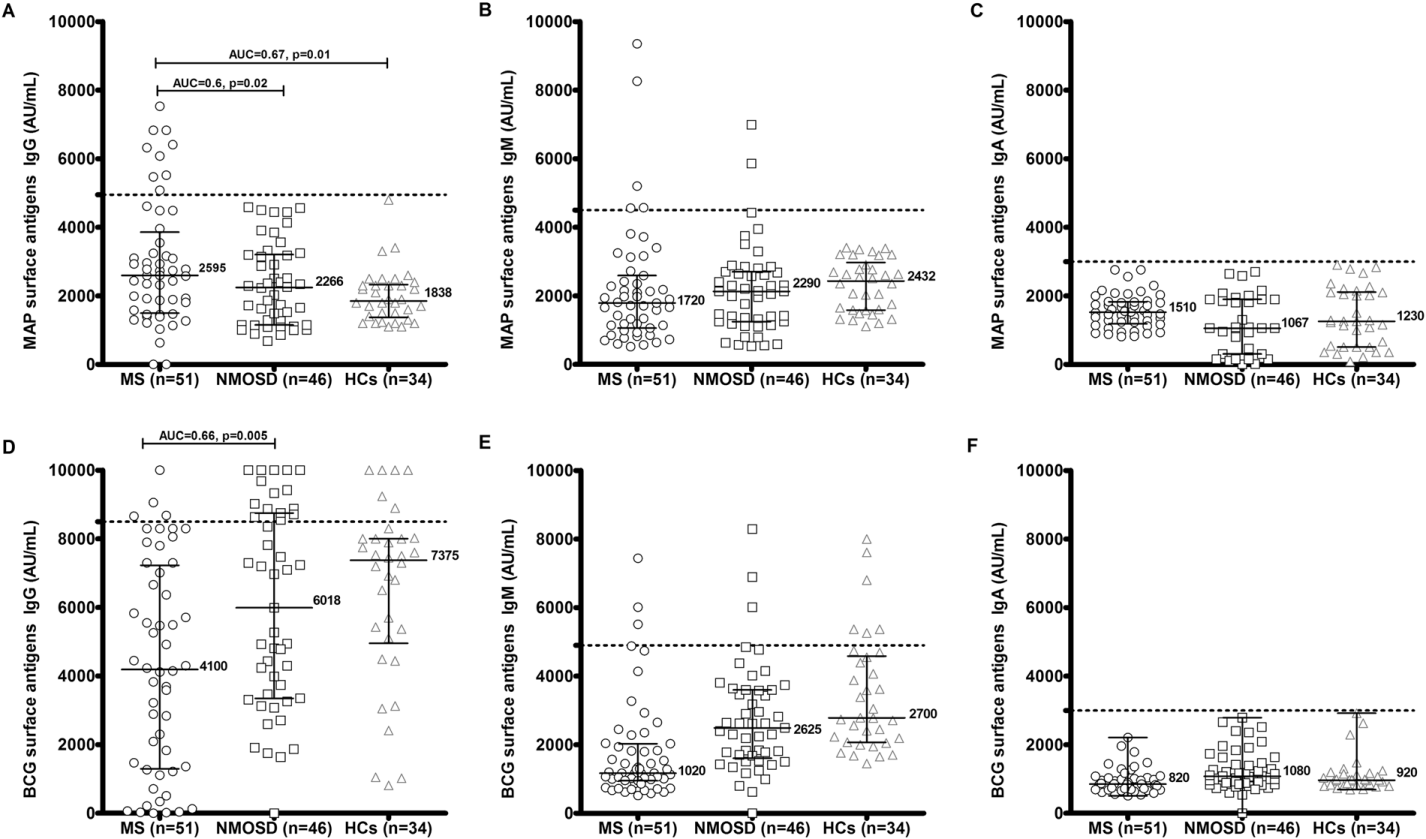
ELISA-based analysis. Fifty-one MS, 46 NMOSD and 34 HCs were screened for Abs reactivity against MAP IgG (**A**), MAP IgM (**B**), MAP IGA (**C**), BCG IgG (**D**), BCG IgM (**E**) and BCG IgA (**F**) by indirect ELISA. The horizontal black bars represent median plus interquartile range, while the dotted lines indicate the cut off for positivity as calculated by ROC analysis. Area under ROC curve (AUC) and P values, significant if <0.05, are indicate by two headed arrows.

Based on the determined cut-off point, 9 out of 51 MS patients (18%, 95% CI: 7.5–28.5%), none of NMOSD patients and none of the HCs were positive for anti-MAP IgG Abs (Fig. 1A). The difference between MS and NMOSD patients, and between MS and HCs was statistically significant (AUC = 0.59, p = 0.02; AUC = 0.67, p = 0.01; respectively).

If we analyze MS clinical characteristic in relation to the IgG-positivity towards MAP, we find that among 9 MAP IgG positive MS patients: high levels of IgG_1_ were observed in 7 [6 RRMS and 1 secondary progressive MS (SPMS)] sera (55.5%, 95% CI: 23–88%), and 2 primary progressive MS (PPMS) sera (22.2%, 95% CI: −5–49%) were positive for IgG_4_. Since IgG_4_ expression is predominantly under the condition of chronic antigenic stimulation^9^, we can hypothesize that an isotype switching from IgG_1_ to IgG_4_ occurred in patients with longer exposure to MAP antigens.

All MAP positive patients were not in relapsing phase at the sampling time. No substantial levels of IgG_2_, IgG_3_, IgM (Fig. 1B) and IgA (Fig. 1C) were detected in all sera.

### Anti-BCG IgG and IgM Abs prevalence is lower in MS patients compared to NMOSD and HCs

Despite all participants were BCG-vaccinated, difference in the humoral responses were observed between the three groups. Anti-BCG IgG Abs were found in 4 out of 51 MS (8%, 95% CI: −1.4–9%), in 15 out of 46 NMOSD (32%, 95% CI: 18.5–45.4%), and in 6 out of 34 HCs (18%, 95% CI: 5.1–30.9%). The difference between NMOSD and MS was statistically significant (AUC = 0.66, p = 0.005) (Fig. 1D). MS positive subjects were RRMS (2), PPMS (1) and SPMS (1). Amongst NMOSD Ab-positive patients, 3 were AQP4-positive and 12 were AQP4 negative. As regards anti-BCG IgM and IgA Abs, there was no statistically significant difference when comparing MS, NMOSD and HCs groups (Fig. 1E,F).

Overall, the Ab titer against BCG lipophilic antigens was elevated but in MS groups, the titer of the majority of subjects falls below the cut-off point.

In a followed up study conducted on 449 Japanese people analyzed before and for 1 year after BCG vaccination, it was detected a general low Ab-response to anti- tuberculous glycolipid antigens Abs in the serum of the vaccinated subjects^10^. Glycolipid antigens are components derived from the cell wall of *Mycobacterium tuberculosis* used for the serodiagnosis of tuberculosis, which are also present in other mycobacteria species included BCG^10^.

Similar to our results, this mechanism might reflect anergy after BCG vaccination. It would be related to the environmental influences such as previous mycobacterial infections, as well as genetic factors^11^.

### Assessment of the MAP and BCG IgG-level in relation to clinical and therapeutic variables

The presence of anti-MAP and anti-BCG Abs was not related to age, age at onset, disease duration, EDSS, disease phenotype, IgG index, oligoclonal bands positivity or the presence of anti-AQP4 Abs. Patients who received oral steroids and those who were treated with steroid pulse therapy were excluded from the study. All 4 MS patients positive for BCG IgG did not received disease-modified immunomodulatory agents at least 6 months before sampling.

Regarding MAP Ab-seropositivity, the mean total serum level in the MS patients was 1128 ± 193 mg/dL, whereas in the NMOSD it was 1266 ± 398 mg/dL (Table 1). Chi square showed that MAP positivity was more frequent in patients under interferon-beta therapy at the time of the blood collection (p = 0.005). Logistic regression confirmed a significant association between the presence of anti-MAP IgG Abs and interferon-beta therapy at study time (OR = 11.9; confidence interval (CI) 95% = 2.2–63; p = 0.004) (Table 2).

**Table 1:** Baseline characteristics of study population.

	MS	NMOSD	HCs
Number	51	46	34
Female/male ratio	38/13 (74%/26%)	28/18 (61%/39%)	25/9 (73%/27%)
Age, years (SD)	41.1 ± 11.3	49.6 ± 15.2	40.0 ± 10.1
Age at onset, years (SD)	32.8 ± 9.8	42.3 ± 15.2	
Duration of disease, years (SD)	8.4 ± 6.8	8.5 ± 12.1	
Oligoclonal bands positivity	31/49 (63%)	8/33 (24%)	
IgG index ≥ 0.7	30/44 (68%)	5/33 (15%)	
Total serum IgG (mg/dL)	1128 ± 193	1266 ± 398	
EDSS score at onset	2.0 (0–7)	2.9 (0–7.5)	

MS, multiple sclerosis; NMOSD, neuromyelitis optica spectrum disorders; HCS, healthy controls; EDSS, Expanded Disability Status Scale.

**Table 2:** Relationship between mycobacterial IgG positivity and interferon-beta (IFN) therapy.

		features		OR	95%CI	p
		**IFN-beta neg**	**IFN-beta pos**	34		
MS (n = 51)	MAP IgG pos	4	5	11.9	2.2–63	0.004*
	MAP IgG neg	38	4			
		**IFN-beta neg**	**IFN-beta pos**			
MS (n = 51)	BCG IgG pos	4	0	1.6	0.1–17	0.6
	BCG IgG neg	39	8			

MS, multiple sclerosis; NMOSD, neuromyelitis optica spectrum disorders; OR, odds ratio; CI, confidence interval; * statistically significant.

When we only considered those MS patients treated with interferon-beta, 5 out of 9 (55%) RRMS patients were MAP IgG positive. These results are in agreement with those of Frau *et al*., who observed a prevalence of total IgG Abs against MAP_2694 protein in 30.7% of MS patients six months after initiating interferon-beta treatment^12^.

The mechanism of interferon-beta in MS and its effects in the modulation of innate and adaptive immune response to pathogens are not fully understood. On the one hand this disease modifying drug seems to enhance the humoral response to MAP^12^. If, however, we consider the mean Ab-titer of the MAP positive MS subjects (n = 9), the interferon-beta-treated patients (n = 5) did not have a statistically significant difference compared to the drug-free ones (n = 4) (6574 ± 747 AU/mL vs 5811 ± 520 AU/mL, p = 0.13), probably because interferon-beta treatment causes a Th1 to Th2 shift or class switching.

### Evaluation of cross-reactivity between anti-MAP and anti-BCG antibody-titers

To determine if the Ab-response observed to MAP surface antigens was specific, 2 MS IgG positive and 2 NMOSD IgM positive sera for MAP were pre-adsorbed with saturating concentrations (titrated for each individual serum) of MAP lipophilic antigen, *Mycobacterium phlei* and BCG lipophilic antigen. Sera were then subjected to ELISA on MAP lipophilic antigen-coated plates.

**Figure 2:**
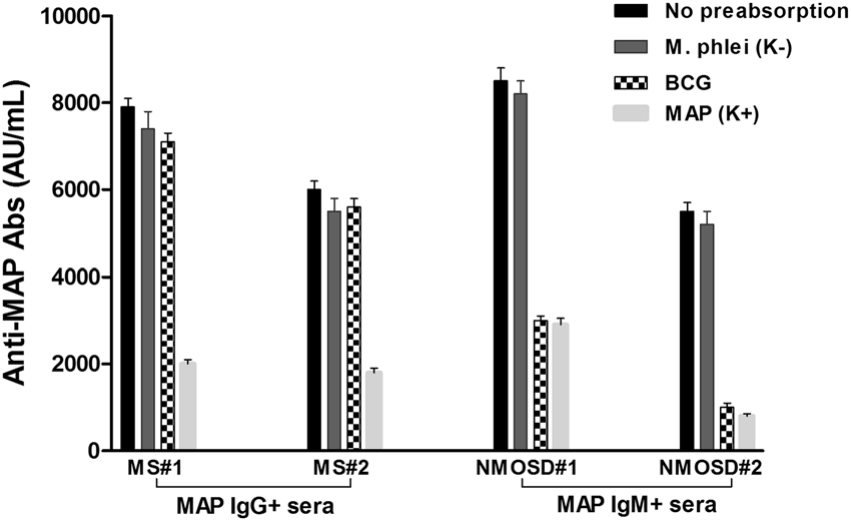
Competition assay. Bars depict means ± standard deviation of triplicate wells and results are representative of two separate experiments.

**Figure 3:**
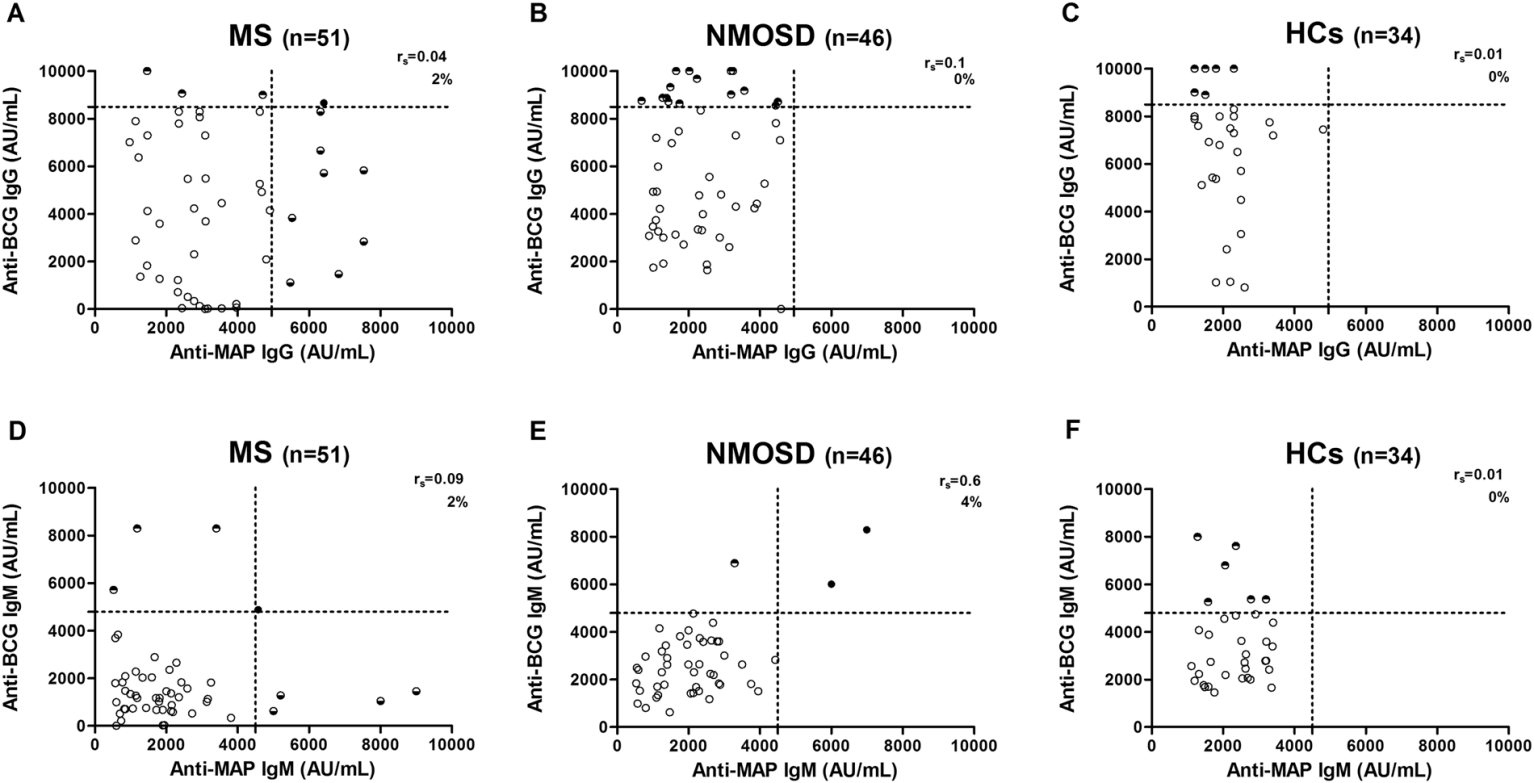
Correlation analysis. Correlation between Abs recognizing anti-MAP and anti-BCG specific IgG in MS (**A**), NMOSD (**B**) and HCs (**C**); and Abs recognizing anti-MAP and anti-BCG specific IgM in MS (**D**), NMOSD (**E**) and HCs (**F**). Each filled black circle represents the titer of a patient with double positivity for MAP and BCG Abs, while each half-filled circle represents the titer of a patients with specie-specific Abs. Empty circles are Ab-negative patients. The dotted line lines designate the cutoff for positivity used in each assay, as calculated by ROC analysis.

In the case of MS sera, both *Mycobacterium phlei* (negative control) and BCG antigens did not cause any decrease in signal, indicating the absence of cross recognition IgG Abs between MAP and BCG (Fig. 2). Besides, regression analysis indicated no correlation between MAP and BCG antigens in MS (r_s_ = 0.04), NMOSD (r_s_ = 0.17) and HCs group (r_s_ = 0.01) (Fig. 3A–C).

Concerning NMOSD subjects, competition experiments showed that nonspecific IgM were elicited by common mycobacterial glycolipid antigens, probably because of the low affinity and cross reactivity of IgM isotype (Fig. 2)^13^. In fact, when pre-adsorbing the sera with BCG antigen, the latter was capable of blocking the binding of the Abs to the targeted MAP antigen-coated, strongly inhibiting the MAP sero-reactivity in the sera (decline in signal between 70–90%) (Fig. 2). Additionally, IgM binding profile of MAP correlate significantly (r_s_ = 0.6) with the profile of BCG lipophilic antigens (Fig. 3E).

## Discussion

T and B cell responses are influenced by the bacterial site in which the antigen is expressed^14^, and bacterial proteins can be expressed in the cytoplasm, bound to the surface of the bacteria or secreted. The majority of the MS-related MAP antigens identified share sequence homology with intracellular human proteins^15–17^. It was hypothesized that the phenomenon of the epitope spreading may contribute to the production of Abs against previously sequestered antigens, leading to a secondary autoimmune response against the newly released intracellular antigens^15^.

In this study we analyzed the humoral response mounted against surface-exposed antigens, which are the primary target of the host immune system^18^.

We found a significantly higher Ab response against MAP IgG Abs in MS patients compared to the other  groups. The main IgG subclass elicited by MAP was IgG_1_, and two MS patients diagnosed with PPMS were positive for IgG_4_ Abs. These findings are congruent with the previous results obtained in a MAP-based seroprevalence study conducted in Japanese subjects, in which elevated IgG_1_ response against MAP lipophilic antigens was detected in the sera of these subjects^8^. However, at present we are unable to establish if MAP is a potential risk factor of conversion from CIS to clinically definite MS, an accelerator of the pathogenesis, or maybe it plays only a peripheral role.

We also evaluated the Ab-prevalence to BCG, and the differences in the Ab-profiles to BCG antigens between the groups were remarkable. BCG antigen was capable to ignite a strong humoral response in NMOSD subjects, while in MS group we observed a low Ab-titer against BCG antigen, indicating that this factor was inversely related to the relative risk of developing MS.

The difference in the anti-BCG and anti- MAP Ab-level between MS and NMOSD groups, may be explained in different ways. First, the different route of transmission/administration of foreign antigens, oral for MAP and intradermal for BCG, might have significant effects in influencing the immune response^19^. Second, it may be that some individuals have, due to a different genetic predisposition, an increased/decreased reactivity to the mycobacterial cell wall-derived antigens. Indeed HLA restriction analysis revealed that depending on the individual’s HLA haplotype, the specific adaptive immune responses to distinct mycobacterial antigens that mimic self-protein could be restricted to HLA products encoded by HLA-DRB1, HLA-DRB3 or HLA-DRB4^20^. Further, it is well known the existence of different genetic and infectious profiles between MS and NMOSD patients in Japan^21, 22^. HLA-DRB1*0405 and HLA-DPB1*0301 were found to be MS susceptibility genes^21^, whereas HLA-DRB1*1602 and HLA-DPB1*0501 conferred susceptibility only to anti-AQP4 antibody-positive NMO^22^. Third, even if there is a considerable overlap in lipid content between MAP and BCG, the low-cross reactivity observed in the competition experiments between anti-MAP and anti-BCG IgG Abs suggests the existence of an antigen-specific humoral immune response. MAP lipopentapetide (L5P) is a specific cell wall component of MAP capable to induce a strong host humoral response, and able to distinguish MAP from other mycobacteria including BCG^23^. A highly specific IgG responses to L5P were detected in patients affected by Crohn’s disease and in children at risk for type 1 diabetes^24, 25^.

Lastly, Ab production is also influenced by interaction between T and B cells and by alteration of cytokine milieu. In NMO there is an enhancement of humoral (Th2) immune response with elevated Il-4 and Il-10 levels compared with levels in MS subjects^26^. Therefore, it is reasonable that the Ab titer against BCG was higher in NMOSD patients. Instead, the higher MAP Ab response in MS compared to NMOSD might be due to CD1 NKT cell recognition. Evidence suggests that clonal population of CD1a-, CD1b- and CD1c restricted T cells specific for mycobacterial lipid antigens can respond differently to MAP and BCG^27^. To note, lipopeptides such as L5P are recognized by CD1a T cells^28^, and polymorphisms of CD1a and CD1e genes have been associated with susceptibility to MS^29^.

In conclusion, the mechanism by which mycobacteria and in particular BCG exert their effect in patients is unknown, and further research is warranted to explore the relationship between Ab prevalence and genotype in MS and NMOSD groups. For example, it will be useful whether serum BCG versus MAP IgG reactivity are able to differentiate MS from NMOSD in the initial attack.

## Methods

### Ethical considerations

The study protocol was approved by the National Ethical Committee of the Juntendo University School of Medicine (Approved No. 205), Sangenjyaya hospital (Approved No. 2014.3.7) and Tohto College of Health Sciences (Approved No. H2511). All the methods were carried out in “accordance” with the approved guidelines. All subjects provided written informed consent for participation.

### Subjects

Baseline characteristics and clinical demographic data of the study population are shown in Table 1. All participating subjects were Japanese and BCG vaccinated. At the time of the sampling, all the subjects were HIV negative and with no history of past tuberculosis (TB) infection.

Fifty-one MS patients fulfilling the revised McDonald diagnostic criteria were enrolled in this study^30^. The clinical characteristic of the MS cases were the following: 46 RRMS, 3 SPMS, 2 PPMS and all AQP4 negative.

Forty-six NMOSD were also included. NMOSD group includes patients with NMO-like symptoms that fulfill the 2015 diagnostic criteria for NMOSD^2^. Thirty-four sex and age-matched healthy volunteers without history of autoimmune diseases were also included as control group.

### Aquaporin 4 cell based assay

AQP4 live transfected human embryonic kidney cells (HEK293) expressing the untagged AQP4-M23 isoform were incubated in 4x diluted serum for 1 h, washed and incubated in fluorescein-conjugated goat anti-human IgG (MP Biomedicals, Aurora, OH, USA) for 30 min. Cells were fixed in 95% ethanol and mounted in the prolong antifade mounted media Permafluor (Beckman Coulter, Fullerton, Ca, USA). Image were captured by confocal microscopy Fluoview (Olympus Tokyo). Ab-seropositivity was based on comparison with mock-transfected cells that did not express AQP4.

### Mycobacterial antigens

Preparation of MAP (strain ATCC 19698) and BCG (strain Tokyo-172) lipophilic antigen was performed as described in detail elsewhere^8^. MAP and BCG surface antigens are a crude extract of the mycobacteria and the lipid fractions were used as an antigen after removing cross-reactive component by absorbing the serum with *Mycobacterium phlei* ^8^. The lyophilized bacteria was obtained from the commercially available ELISA kit, Johnelisa II kit (Kyoritsu Seiyaku Corporation, Tokyo, Japan).

### ELISA

Optimal conditions for the indirect ELISAs were established by titration experiment following our previously published methodology^8^. BSA-coated wells were included as a negative control, and the mean value obtained was subtracted from all other data points. A serum sample from a patient with high IgG anti-MAP was included in all experiments and used as the internal laboratory positive control. All test serum samples were therefore normalized against this sample, the reactivity of which was set at 10,000 arbitrary units (AU)/mL. Experimental samples were analyzed in triplicate.

### Inhibition assay

In order to assess potential cross-reactivity between mycobacterial Abs, a competitive ELISA was performed. Briefly, diluted MAP Ab-positive serum samples were pre-absorbed with saturating concentrations [10–15 mM] of BCG lipophilic antigen for 2 h at room temperature, and then sample where transferred in plates coated with MAP lipophilic antigens and subjected to indirect ELISA as previously described^8, 15^. MAP lipophilic antigen was used as a positive control for a true Ab interaction, and *Mycobacterium phlei* antigen was applied as negative control for nonspecific reactions.

### Statistical analysis

Statistical analysis was performed using Graphpad Prism 6.0 software (San Diego, CA, USA). Receiver operating characteristic (ROC) curves were used to evaluate the diagnostic accuracy of the ELISA to detect specific Abs against the mycobacterial antigens. Comparison of the ELISA results between patient and HCs was statistically analyzed using Mann-Whitney’s nonparametric, unpaired, two-tailed test, Chi square and Fisher’s exact probability test. Spearman correlation analysis was performed in order to test cross-reactivity between mycobacterial Abs. Logistic regression-derived odds ratio (OR) was used to evaluate Ab-positivity with respect to clinical independent variables.
