# Author Correction: Altered humoral immunity to mycobacterial antigens in Japanese patients affected by inflammatory demyelinating diseases of the central nervous system

**DOI:** 10.1038/s41598-020-59760-3

**Published:** 2020-02-11

**Authors:** Davide Cossu, Kazumasa Yokoyama, Yuji Tomizawa, Eiichi Momotani, Nobutaka Hattori

**Affiliations:** 1 Juntendo University School of Medicine, Department of Neurology, Tokyo, 113-8421 Japan; 2 Tohto College of Health Sciences, Department of Human-care, Saitama, 366-0052 Japan

Correction to: *Scientific Reports* 10.1038/s41598-017-03370-z, published online 09 June 2017

The Acknowledgements section in this Article is incomplete.

“Davide Cossu is an “overseas researcher under Postdoctoral Fellowship of Japan Society for the Promotion of Sciences”.”

should read:

“Davide Cossu is an “overseas researcher under Postdoctoral Fellowship of Japan Society for the Promotion of Sciences”. Yokoyama Kazumasa and Hattori Nobutaka research studies are financially supported by Ono Pharmaceutical, Mitsubishi Tanabe Pharma, Hydrogen Hearlth Medical Labo, ABIST, Melodian, Daiwa, Biogen Idec Japan, Bayer Yakuhin, Nihon Pharmaceutical, Asahi Kasei Medical, MiZ and Kenkokazoku. All Authors report no competing interest.”

